# Update on the Management of Acute Sigmoid Volvulus: Endoscopic Decompression and Definitive Surgical Treatment

**DOI:** 10.7759/cureus.116789

**Published:** 2026-09-23

**Authors:** Constantinos Avgoustou, Christina Avgoustou, Konstantinos Skevis, Dimitrios Jannusis

**Affiliations:** 1 Department of Surgery, General Hospital of Nea Ionia “Konstantopouleio”, Athens, GRC; 2 Fourth Department of Internal Medicine, National and Kapodistrian University of Athens, Athens, GRC; 3 Department of Thoracic Surgery, “Sismanoglio – Amalia Fleming” General Hospital of Attica, Athens, GRC; 4 Department of Air Ambulance Services, Hellenic National Center of Emergency Care (EKAB), Athens, GRC

**Keywords:** bowel obstruction, endoscopic decompression, endoscopic detorsion, gangrenous sigmoid, sigmoid resection, sigmoid volvulus

## Abstract

Sigmoid volvulus (SV) is a form of mechanical bowel obstruction with a likely multifactorial etiology. It occurs far more frequently in the so-called “volvulus belt” countries than in Western nations. In Western countries, its relatively low incidence has led to a lack of management experience. Establishing a diagnosis can be difficult and challenging, while the timing of presentation is a crucial determinant of prognosis. Delayed admission may result in bowel ischemia and gangrene. These complications are associated with increased morbidity and high mortality. Many aspects of SV management remain debatable, and proposed treatment algorithms are not universally accepted. In most cases of acute, uncomplicated SV, endoscopic decompression is the first-line treatment. However, this approach is commonly associated with SV recurrence if it is not followed by surgery. For complicated SV cases, emergent resection surgery can provide an effective and definitive treatment. Elderly patients and those with severe comorbidities may require careful preoperative assessment and optimization. Definitive surgery under general anesthesia is often postponed in this high-risk population. This review aims to examine the diagnostic challenges associated with acute SV and to evaluate the safety and effectiveness of currently available therapies. Further clinical research is needed to clarify the most appropriate treatment strategies for SV.

## Introduction and background

Sigmoid volvulus (SV) is defined as the abnormal twisting of the sigmoid colon around its mesentery. It represents an important cause of mechanical large bowel obstruction. In Western countries, it is the third most common cause of large bowel obstruction, following colorectal cancer and complicated diverticular disease. Conversely, in the endemic “volvulus belt” region, it accounts for approximately 50% to 80% of all large bowel obstruction cases [1-8].

Acute SV constitutes a true closed-loop intestinal luminal obstruction. It can have a substantial impact on patient morbidity, mortality, and healthcare expenditures, while it also adds a significant burden to emergency surgical care [1,4,9-11]. Considerable geographic variations in disease incidence and patient demographics have resulted in heterogeneous management practices worldwide. Consequently, universally accepted treatment guidelines remain lacking [5]. Additionally, the relatively low incidence of SV in Western populations has led to a lack of experience in managing this condition [7].

The objectives of this review are twofold. First, we aim to provide a comprehensive overview of the current understanding of the SV form of colonic volvulus. Second, we seek to delineate the spectrum of available management strategies for SV, ranging from conservative measures to definitive surgical interventions.

## Review

Methodology

A comprehensive literature search was executed in June 2026 across the MEDLINE, PubMed, and Embase databases. The literature search encompassed articles published from 2019 through May 2026. The review aims to examine the diagnostic challenges associated with SV and to evaluate the safety and effectiveness of currently available therapies. Studies were critically evaluated based on their disease definitions. Study selection was strictly restricted to studies employing rigorous and verifiable diagnostic criteria for SV, including clear radiologic, endoscopic, and intraoperative confirmation. Eligible articles were required to report on reasonable treatments using adequate sample sizes, specifically including more than 20 patients.

The following exact Boolean search strings were deployed during the electronic query: (“Sigmoid Volvulus” OR “SV”) AND (“Management” OR “Approach” OR “Algorithm” OR “Treatment”); “Sigmoid Volvulus” AND “Epidemiology”; “Sigmoid Volvulus” AND “Etiology”; “Sigmoid Volvulus” AND “Pathophysiology”; “Sigmoid Volvulus” AND “Diagnosis”; “Sigmoid Volvulus” AND (“Surgery” OR “Surgical procedure”); “Sigmoid Volvulus” AND (“Endoscopy” OR “Endoscopic detorsion”). Other terms closely related to SV, namely, “bowel obstruction” and “gangrenous sigmoid,” were also utilized. Literature selection was governed by predefined, objective inclusion and exclusion criteria.

Inclusion criteria consisted of (1) original, peer-reviewed investigations (including prospective cohorts, retrospective cohort studies, and case series containing more than 20 patients); (2) clinical studies evaluating adult patient populations diagnosed with SV or cecal volvulus (CV); (3) studies published exclusively in the English language; and (4) research conducted entirely on human subjects.

Exclusion criteria included (1) anecdotal case reports (comprising fewer than 20 patients), conference abstracts/proceedings, editorial correspondence, systematic reviews, meta-analyses, and expert commentary; (2) non-English language publications; and (3) animal models, preclinical studies, or in vitro experimental designs.

Title and abstract screening of all electronically retrieved citations was performed independently by two reviewers against the eligibility criteria. Any disagreements regarding study inclusion were resolved via collaborative consensus. Data extraction focused on study design, sample size, clinical management algorithms, and surgical modalities, followed by a qualitative synthesis within this narrative review. The reference lists of all eligible articles underwent secondary manual citation tracking. This manual cross-referencing allowed for the targeted inclusion of highly illustrative, landmark papers and select reviews that provided critical clinical insights into optimal clinical management and evolving therapeutic paradigms. Particular attention was given to multidisciplinary management and the personalized application of treatment options.

Unpublished images from our own material are used in the diagnosis section. The images were retrospectively retrieved from the imaging database of General Hospital of Nea Ionia “Konstantopouleio”, Athens, Greece, between November 2010 and May 2012.

Epidemiology

The incidence of colonic volvulus varies considerably across different geographic regions. Generally, it is higher in developing countries [2,3]. In the so-called “volvulus belt,” which encompasses areas such as Africa, the Middle East, Eastern Europe, Russia, India, and South America, volvulus represents 13% to 42% of all intestinal obstructions [5,12]. Within Eastern Europe, Africa, and India, SV alone is responsible for nearly 50% of intestinal obstructions [2,5,13,14]. Conversely, volvulus accounts for only 10% to 15% of all large bowel obstructions in the United States and Western Europe. Specifically, SV accounts for approximately 4% of all large bowel obstruction cases in the United States and the United Kingdom [2-5,13,15]. The estimated annual incidence of SV is 1.67 cases per 100,000 individuals in the United States, compared with 4.2 per 100,000 in Eastern Anatolia [8].

In countries within the “volvulus belt,” SV typically affects young men from the fourth decade onward. In these regions, the male-to-female sex ratio is 4:1 [5]. The lower incidence among females has been attributed to anatomical differences. These include a more capacious pelvis with lax musculature, which may facilitate the spontaneous untwisting of a redundant sigmoid colon [15]. In contrast, SV in Western countries predominantly affects elderly males, usually older than 70 years. These patients often have multiple comorbidities, such as constipation and reduced mobility. By comparison, CV tends to affect a somewhat younger female population, typically under 60 years of age [5,7,10,12,16].

Pregnancy constitutes a distinct population in the management of SV. According to the literature, SV is among the most common types of intestinal obstruction in pregnant women, accounting for approximately 44% of all cases [8,17].

Although any mobile colonic segment can volvulize on itself, the sigmoid colon is involved in 60% to 75% of cases in most series. The cecum is implicated in 25% to 40% of cases. Conversely, the transverse colon and splenic flexure are much less commonly affected, accounting for approximately 1% to 4% and 1% of cases, respectively [5,12,18].

Contemporary cohort studies of SV collect demographic, clinical management, and outcome data. These studies include measures of frailty as well as risk stratification tools, such as the American Society of Anesthesiologists (ASA) score and the National Emergency Laparotomy Audit (NELA) score. Utilizing these tools helps better evaluate patient outcomes and guide clinical decision-making [19].

Etiology

The etiology of volvulus, which is generally an independent benign condition, is probably multifactorial. However, current evidence does not clearly identify any single causative or predisposing factor [2,5,20]. Some risk factors are common to all locations of the disease. These include chronic constipation, the frequent use of laxatives or specific enemas, a high-fiber diet, previous abdominal surgery, and underlying anatomical predispositions [2,5,12].

The most commonly cited predisposing factor for SV, directly related to anatomy, is the dolichosigmoid. This condition is characterized by an excessively elongated sigmoid colon on a narrow mesenteric attachment [2,7,12,21,22]. This anatomical configuration formed the basis of the classical mechanism of volvulus proposed by Ballantyne in 1982. In this model, a redundant sigmoid colon twists around a narrow mesenteric pedicle [21]. Radiological studies using barium enema by Madiba et al. [22] supported this theory. They demonstrated the role of the markedly elongated sigmoid colon and mesosigmoid in African patients with SV. Similarly, Bhatnagar et al. [23] studied sigmoid anatomy. They postulated that individuals with a dolichomesocolic sigmoid colon, in which the mesocolon is longer than it is wide, are more susceptible to SV compared to individuals with a brachymesocolic sigmoid colon, where the mesocolon is wider than it is long, who are less susceptible. More recently, Akinkuotu et al. [24] challenged aspects of Ballantyne’s hypothesis in a case-control study. They proposed that a long and wide mesosigmoid combined with a narrow mesenteric root is the key anatomical configuration predisposing to SV. However, although anatomical abnormalities clearly play a central role in the development of SV, it remains uncertain whether these changes are congenital, acquired, or the result of interactions between both factors [5,12,15,24]. Nevertheless, available evidence suggests that SV is predominantly an acquired condition. Conversely, volvulus of the right colon is typically congenital in origin [20].

Several additional factors have been implicated in the pathogenesis of SV. These include diabetes mellitus, neuropsychiatric disorders associated with reduced mobility and autonomy, and institutionalization [2,12,24]. In younger patients, SV is frequently associated with megacolon secondary to conditions such as Hirschsprung’s disease or Chagas disease [3,5,12,18]. Less commonly, SV may occur in association with complicated midgut malrotation [25] or with sigmoid or rectal carcinoma [12,26]. The coexistence of SV and colorectal cancer is exceptionally rare. It has been only sporadically reported in the literature [26]. Nevertheless, the presence of an underlying malignancy has important therapeutic implications and modifies surgical management, as it necessitates an oncological resection in accordance with cancer surgery principles.

Pathophysiology

Acute SV results from the axial rotation (torsion) of a redundant sigmoid colon around its mesenteric axis [2]. For unknown reasons, the twist preferentially occurs in a counterclockwise direction in approximately 70% of cases [4,5]. The two main mechanisms of torsion in SV are believed to be either axial mesocolic volvulus (75% of the time) or organoaxial volvulus (25% of the time) [5]. Persistent torsion beyond 180 degrees results in colonic luminal occlusion. This may lead to bowel ischemia, necrosis, and perforation. Spontaneous detorsion occurs in only 2% of cases [4,12]. Based on the rapidity of onset, degree, and intensity of twisting, SV can be classified into the following three categories: subacute, acute, and fulminant [3]. The subacute form is the most common and is often characterized by a more indolent clinical course. However, serious complications may still develop over time [3]. Recurrent episodes of SV can induce fibrosis and the progressive shortening of the sigmoid mesentery, which promotes future volvulus events [8].

During severe SV, the enormous colonic distension is associated with a substantial rise in proximal colonic intraluminal pressure. This rise results in impaired capillary perfusion [5,12,27]. Venous obstruction occurs initially, followed by arterial compromise. Concomitant occlusion of the mesocolic vessels further exacerbates colonic mural ischemia [5,27]. Early mucosal ischemia promotes bacterial translocation and intraluminal gas production. This process further increases colonic distension, metabolic toxic derangements (such as acidosis with dehydration), and the development of sepsis [5]. As the disease progresses, patients may develop multiple organ dysfunction syndrome (MODS) and, ultimately, multiple organ failure (MOF) [4,5]. If colonic torsion is not promptly reversed, it results in ischemic gangrene, reperfusion injury, and colonic perforation. Consequently, the patient’s state deteriorates into mixed septic and cardiovascular shock [5,12]. The incidence of bowel gangrene has been reported as 56.3% by Deresse et al. [11] and 18.5% by Moro-Valdezate et al. [28] in relatively large patient series.

In contrast to SV, acute colonic pseudo-obstruction (ACPO), also known as Ogilvie syndrome, is a functional rather than mechanical disorder, associated with a significant overall mortality rate. Although its pathogenesis remains incompletely understood, the prevailing hypothesis involves the altered regulation of gastrointestinal and colonic motility by the autonomic nervous system. This results in significant colonic dilatation in the absence of a demonstrable mechanical blockage [2,14,29].

Diagnostic evaluation

Volvulus typically presents with nonspecific clinical symptoms [12]. According to the World Society of Emergency Surgery (WSES), symptom duration may range from a few hours to several days [5]. In SV, delayed presentation is not the sole determinant necessitating emergency surgery. Other factors, including the degree of torsion (e.g., over-rotation) or individual variations in mesenteric vascular anatomy, may trigger bowel ischemia and gangrene. Notably, these complications can occur even after a relatively short symptomatic period [8].

Following a thorough medical history and comprehensive physical examination, clinically suspected acute SV should be confirmed through appropriate radiological imaging and laboratory investigations [5,10]. Prompt multidisciplinary assessment and management are essential. This is particularly true for emergent, critically ill patients in whom the diagnosis remains uncertain.

Clinical Symptomatology and Signs

Acute SV can present with the classic clinical triad of abdominal pain or tenderness, abdominal distension, and constipation or obstipation [8,10,12]. In patients from the “volvulus belt” regions, the disease may present in a fulminant form characterized by peritonitis and shock. In such cases, the prognosis is often poor because colonic necrosis and perforation have usually already occurred [12].

The clinical spectrum of SV is broad, ranging from nearly asymptomatic disease to generalized peritonitis [8,19]. In Western countries, SV frequently follows a recurrent pattern. Each episode carries the potential for significant morbidity and mortality [5]. Although both specific and nonspecific symptoms may be present, prompt diagnosis and effective treatment are imperative due to the risk of intestinal damage and death [5].

Abdominal pain, often accompanied by tenderness, is one of the hallmark clinical features of SV. However, pain alone does not constitute an absolute indication for emergency surgery [8]. In contrast, rebound tenderness and muscular rigidity are alarming and highly concerning findings. These signs suggest an acute abdomen and generally warrant emergency surgical intervention [8,12,30].

Abdominal distension is another prominent clinical feature of SV. It is frequently associated with several days of colonic obstruction, particularly in Western countries [8,12,30]. Classically, asymmetric gaseous abdominal distension, accompanied by relative emptiness in the left iliac fossa, is considered highly suggestive of SV [12]. However, excessive distension alone is not an absolute indication for emergency surgery, although it may hinder successful endoscopic detorsion [8]. The absence of signs of peritoneal irritation is common in Western countries, which can contribute to delays in diagnosis [8,12].

Constipation or obstipation represents the third major clinical feature of SV. It typically manifests as an absolute inability to pass feces and flatus in acutely affected patients [8,30]. In some patients, residual fecal material and gas distal to the obstruction site may permit a limited number of forceful bowel movements. This can result in limited defecation and flatus despite complete proximal obstruction [8]. Diarrhea has occasionally been reported as a premorbid symptom triggering the onset of volvulus [8]. On digital rectal examination, an empty rectum is the most common finding. However, the presence of blood-stained stool or “gangrenous stool” (hematochezia) strongly suggests bowel ischemia. This finding constitutes an absolute indication for emergency surgery, particularly in delayed presentations [8,10,11,28,30].

The incidence of vomiting increases with prolonged obstruction due to the accumulation of gastrointestinal content. Although vomiting is not an absolute indication for surgery, its presence often reflects disease progression and increases the likelihood that emergency intervention will be required [8]. Hyperactive bowel sounds, such as hyperkinetic or hypertympanic sounds, are common findings in uncomplicated SV. In contrast, as the bowel becomes fatigued, these findings are replaced by hypoactive or absent bowel sounds. This change indicates advancing obstruction and impending bowel compromise, serving as a relative precursor to emergency surgery [8,10].

The prognosis of gangrenous SV is considerably worse compared with the non-gangrenous disease [8]. Toxic, septic, or hypovolemic shock is frequently seen in delayed SV cases or cases with bowel gangrene. All of these scenarios necessitate prompt medical support and emergency surgery [8]. Reported rates of shock range from 9.8% in the series by Moro-Valdezate et al. [28] to 11.7% in the study by Deresse et al. [11] and 15.1% in the work by Pattanaik et al. [30]. Reported mortality rates range from 17% to 100% in patients with gangrenous SV, compared with 10% to 30% in non-gangrenous cases [4].

Pregnancy-associated SV represents a particularly challenging surgical emergency, where diagnosis may be delayed because presenting symptoms frequently mimic common obstetric conditions. Consequently, rapid recognition and optimal treatment in an appropriate healthcare center are essential, as this condition may provoke preterm labor and adversely affect both maternal and fetal outcomes [17,31]. In selected cases, gestation may be safely preserved while definitive treatment of SV is undertaken [17]. Beyond obstetric populations, SV must also be differentiated from ACPO, which mostly affects elderly, debilitated, and hospitalized patients [2,14,29]. Furthermore, in patients presenting with SV, underlying malignancy can be ruled out with an abdominal/pelvic computed tomography (CT) or during colonoscopy [5,12,26].

Laboratory Assessment

Laboratory findings primarily reflect the physiological consequences of bowel obstruction, sepsis, or, less commonly, gastrointestinal bleeding. These findings do not point specifically to the diagnosis [12]. However, laboratory results can provide valuable information regarding disease severity and the patient’s overall clinical status. Following clinical suspicion, routine investigations should be performed. These include a complete blood count, liver and renal function tests, inflammatory markers, serum electrolytes, a coagulation profile, and an arterial blood gas test. When interpreted alongside radiological imaging and, when indicated, endoscopic findings, these investigations contribute to confirming the diagnosis and guiding management decisions [10].

Several clinical scoring systems are commonly employed to assess disease severity, perioperative risk, and prognosis in patients with SV. These include the Sequential Organ Failure Assessment (SOFA) score and the Acute Physiology and Chronic Health Evaluation II (APACHE II) score, as well as the ASA classification system (with most patients classified in ASA groups IV-V) and the NELA score [3-5,8,19,32]. The occurrence of MODS and MOF is also frequently documented because of their strong association with adverse outcomes [4]. Large clinical databases, such as the American College of Surgeons National Surgical Quality Improvement Program (ACS NSQIP), can provide valuable data for retrospective analyses and outcome studies in patients undergoing surgery for SV [33].

Imaging

Initial plain abdominal radiographs are frequently diagnostic of SV (Figures 1, 2). Typical findings include evidence of a closed-loop large bowel obstruction with multiple air-fluid levels. Characteristic radiographic signs are also present, including the “coffee bean” sign, which represents a massively dilated sigmoid loop extending toward the upper abdomen, often above the level of the transverse colon [5,13,34]. Based on these findings, the WSES consensus guidelines on SV management provide a strong recommendation for using abdominal radiography as an initial diagnostic tool in suspected cases [5].

**Figure 1:**
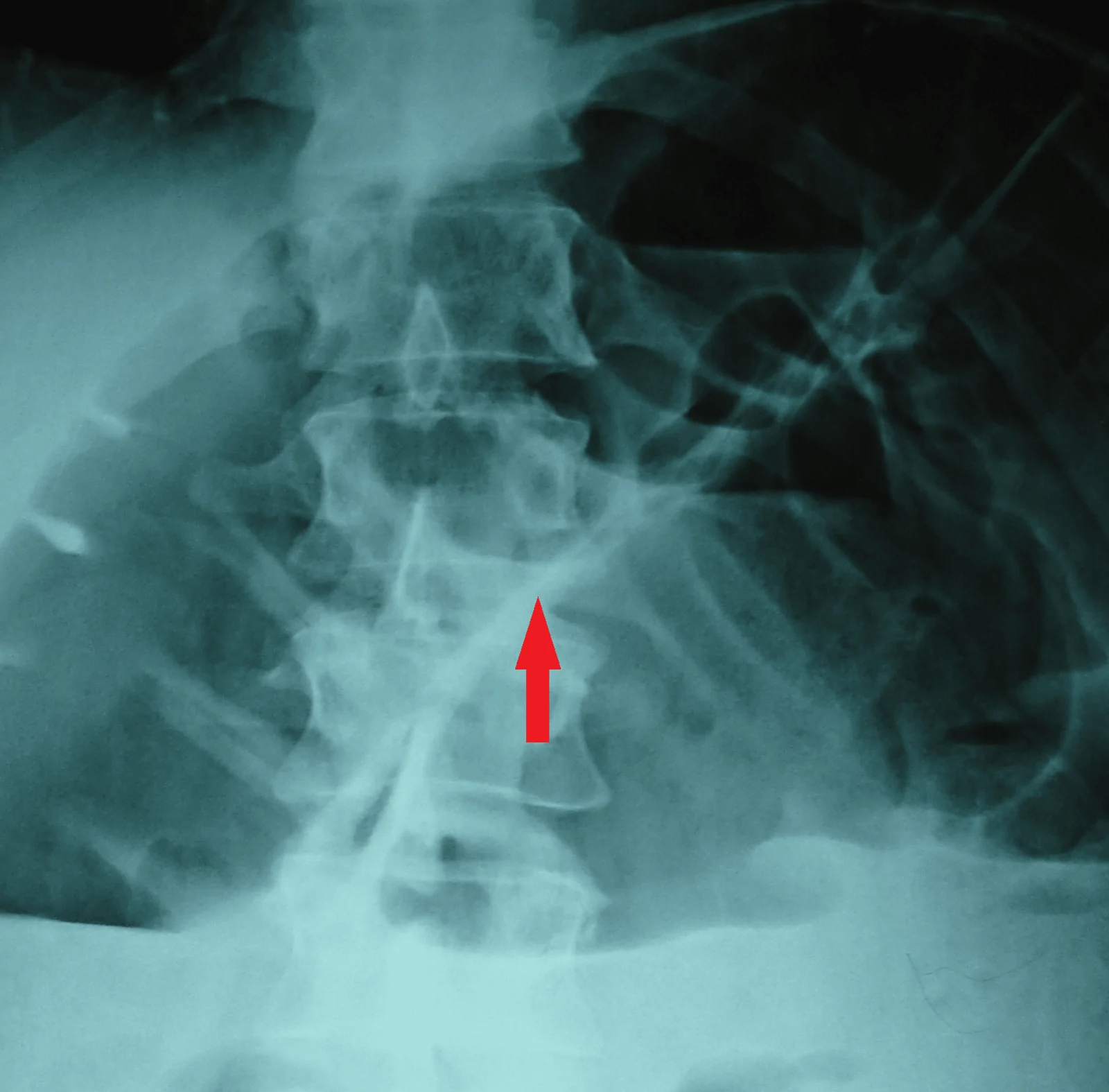
Abdominal radiograph demonstrating sigmoid volvulus (erect view). Abdominal radiograph demonstrating sigmoid volvulus, showing closed-loop obstruction with massive accumulation of air within the colon. The arrow indicates the “unified” wall of two adjacent bowel loops.

**Figure 2:**
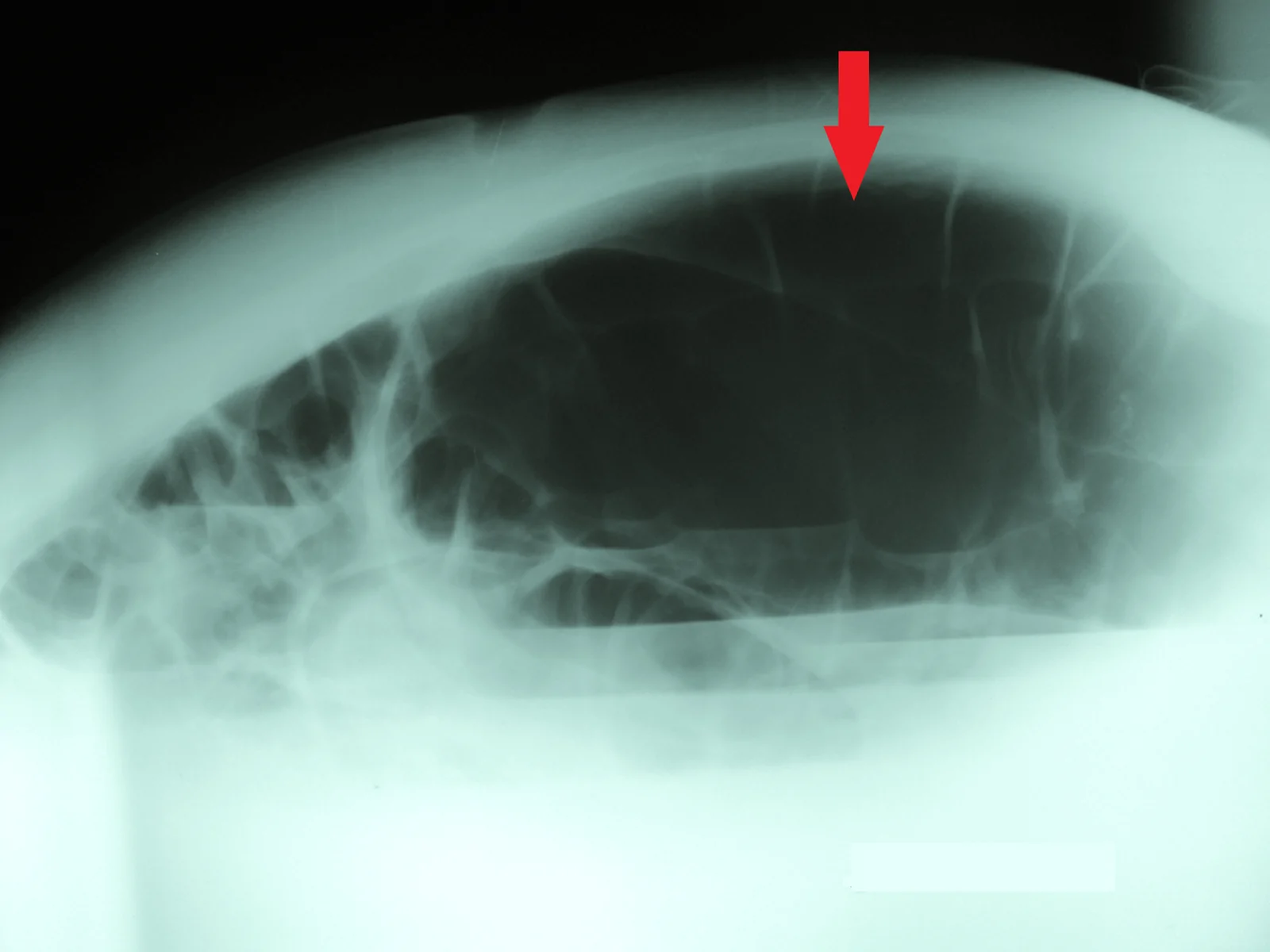
Abdominal radiograph demonstrating sigmoid volvulus (decubitus view). The abdominal radiograph demonstrates a sigmoid volvulus characterized by a massive, gaseous closed-loop obstruction. The red arrow indicates the enormous colonic dilation.

CT scan is the preferred imaging modality for diagnosing cases of colonic volvulus, including SV. It demonstrates nearly 100% sensitivity and greater than 90% specificity [2,5,12]. A CT scan allows for multiplanar image reconstruction, facilitating an accurate and comprehensive assessment of disease severity. Furthermore, it can identify critical complications, such as impending ischemia, bowel necrosis, perforation, pneumatosis intestinalis, and the extent of colonic distension (Figures 3, 4). CT is also valuable in excluding alternative conditions from the differential diagnosis. It particularly helps rule out obstructing colorectal carcinoma at the rectosigmoid junction and ACPO [2,5,12].

**Figure 3:**
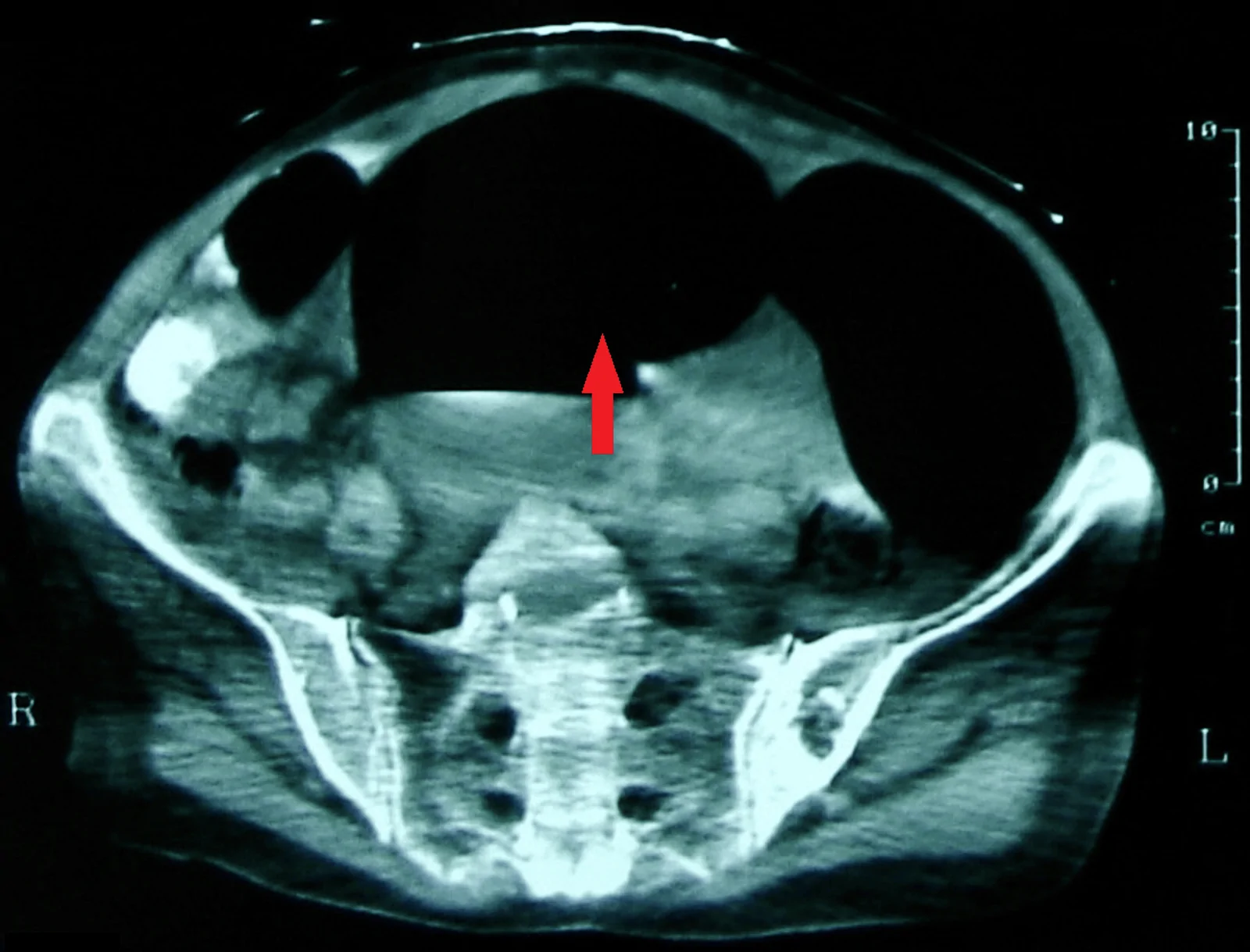
CT scan profile of sigmoid volvulus (axial view). Axial CT scan demonstrating sigmoid volvulus. The red arrow indicates the site of severe, disproportionate dilation of a sigmoid loop with entrapment of a massive volume of air, characteristic of a closed-loop obstruction.

**Figure 4:**
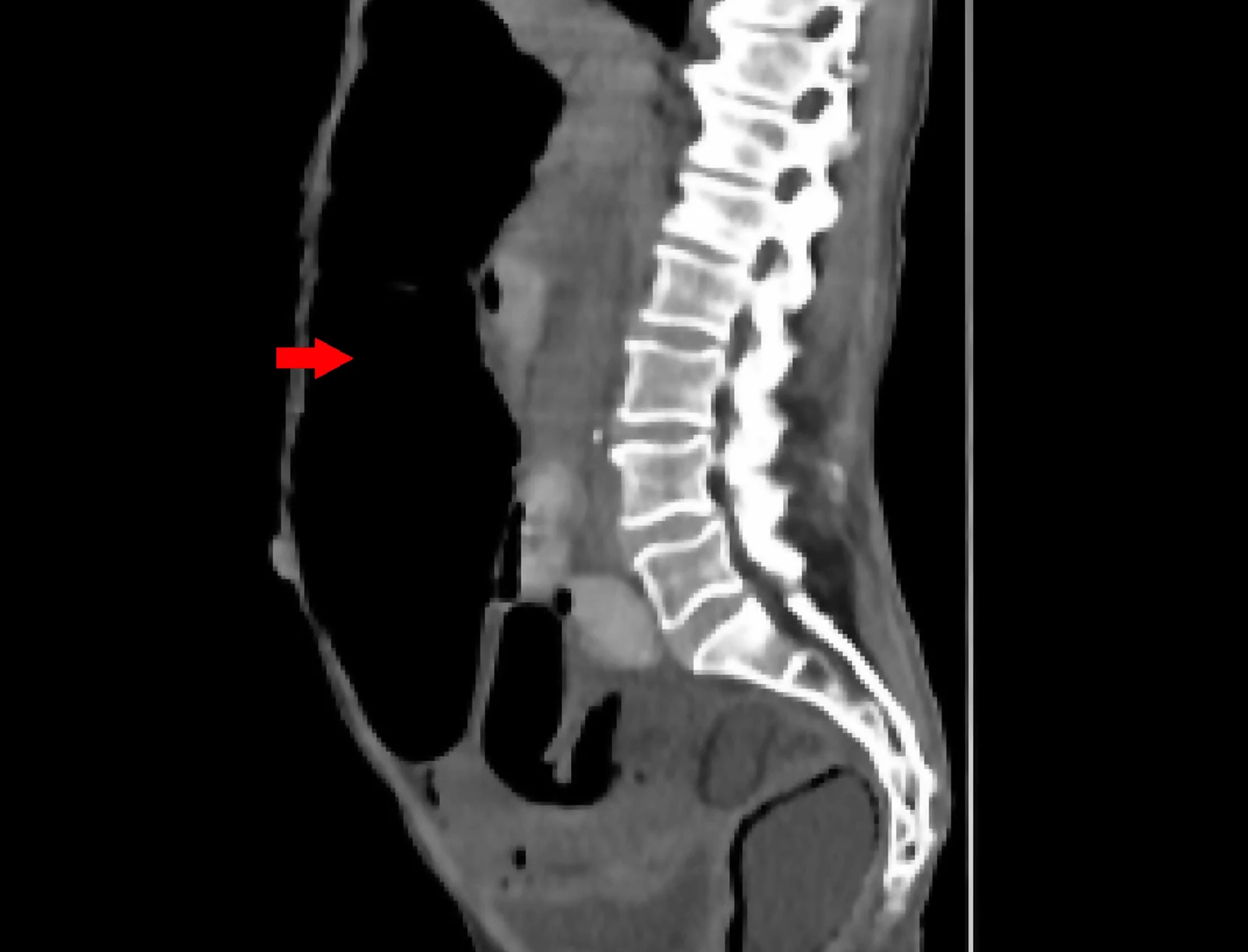
CT scan profile of sigmoid volvulus (sagittal view). The arrow indicates massive accumulation of air within the colon. Sagittal CT scan of sigmoid volvulus, clearly illustrating the massive craniocaudal extension of the disproportionately dilated sigmoid loop, occupying most of the anterior abdominal cavity. The red arrow points to the severe loop dilation and luminal gas entrapment.

Contrast enema studies can further support the diagnosis by demonstrating the characteristic “bird’s beak” sign at the site of colonic torsion. Notably, water-soluble contrast agents are preferred over barium contrast. This preference reduces the risk of chemical peritonitis in cases where the colon is already perforated [5,12,13]. Another classic finding described on CT is the “whirlpool” sign. This sign corresponds to the point of torsion around which the bowel loops and mesenteric vessels are wrapped, making it highly suggestive of volvulus [2,12,25,35]. Additional indicators include the “X-marks-the-spot sign,” which corresponds to the crossing of the two limbs at the common transition point, and the “split wall sign,” resulting from the sigmoid loop wrapping around its mesentery [12]. Finally, the well-known “coffee bean” sign may be visualized on CT imaging, representing the markedly distended and twisted sigmoid loop [12].

Magnetic resonance imaging (MRI) generally offers a diagnostic performance approaching that of CT. It can serve as a radiation-free alternative in specific populations, such as pregnant or pediatric patients [5,36]. However, MRI is often less suitable in acute settings, particularly for debilitated patients suffering from restlessness and dyspnea, as it requires longer scan times and is highly susceptible to motion artifacts [36]. In addition, the utility of MRI remains limited by high costs and restricted availability.

Artificial Intelligence

Traditional diagnostic methods in emergencies rely strictly on clinical expertise, supplemented by imaging interpretation and laboratory evaluation. Consequently, diagnostic accuracy and appropriate management decisions depend heavily on physician experience and the availability of multidisciplinary consultation. In resource-limited settings, access to critical care may be restricted, which can subsequently delay optimal management. Artificial intelligence (AI) applications in the diagnosis, treatment, and surveillance of various medical conditions have expanded rapidly to facilitate clinical decision-making and provide valuable clinical support. AI systems, such as GPT-4V, Med-PaLM, BioGPT, and multimodal LLaMA architectures, integrate specialized models for radiology, emergency medicine, intensive care, and surgical decision-making. Through this integration, they can achieve guideline concordance approaching human expert-level practice in intestinal ileus and volvulus management.

In support of this, a large recent study [37] comprising 234 adult patients demonstrated that multidisciplinary AI systems can achieve high diagnostic accuracy, indicating that focused AI integration can provide valuable clinical support. However, this study also acknowledged several limitations, including the quality of input data, which warrant careful consideration.

Currently, the body of related literature remains limited, and the deployment of AI platforms in emergency settings is restricted due to inherent technical and clinical challenges. Specifically, critical translational hurdles, including the risk of model hallucinations, limited image explainability, and unresolved medicolegal liabilities during acute surgical triage, must be thoroughly addressed. Therefore, further prospective validation studies are warranted to establish their clinical utility.

Treatment

Preoperative medical support for SV focuses on urgent hemodynamic stabilization and aggressive fluid resuscitation to achieve safe bowel decompression before definitive surgical intervention [5,12]. When required, particularly in advanced cases, intravenous correction of fluid deficits and electrolyte imbalances is initiated immediately. Furthermore, administering broad-spectrum antibiotics is essential. These agents must cover both aerobic and anaerobic microorganisms to prevent or control bacterial translocation across the potentially ischemic intestinal wall [5]. Monitoring urine output and central venous pressure is also required for frail or hemodynamically unstable patients.

The therapeutic strategy for SV is determined by several factors. These include the specific anatomical location of the volvulus, the clinical presentation, and the duration of symptoms. Other critical factors include the patient’s physiological status, initial laboratory and imaging findings, and the presence of significant comorbidities [5,8,10,12,27]. To ensure optimal outcomes, the primary objectives are the early identification of bowel viability and the prompt recognition of bowel ischemia, gangrene, or perforation. Effective relief of obstruction, eradication of sepsis when present, and the prevention of recurrence are also vital [4,5,7,8].

Despite advances in diagnosis and treatment, the algorithm for optimal SV management remains a matter of debate, and no universally applicable strategy has been adopted [1,28]. In 2023, the WSES [5] convened a worldwide panel of medical experts to review current evidence and propose consensus recommendations for the diagnosis and management of SV. Accordingly, the widely accepted principal strategy consists of early endoscopic detorsion and decompression, followed by elective surgery in uncomplicated cases. Conversely, emergency surgical treatment is reserved for patients presenting with bowel ischemia, gangrene, perforation, or peritonitis, as well as for unsuccessful endoscopic detorsion, early recurrence, or diagnostic uncertainty [5,10,12,38,39]. Similarly, the American Society of Gastrointestinal Endoscopy (ASGE) has published guidelines regarding the role of endoscopy in managing both SV and ACPO [2].

Surgical management of SV can be broadly divided into resectional and non-resectional procedures. Importantly, operative management is associated with substantial morbidity and mortality, particularly among elderly patients and those with multiple comorbidities [10,15].

Figure 5 is a management algorithm that summarizes treatment options in relation to the patient’s performance.

**Figure 5:**
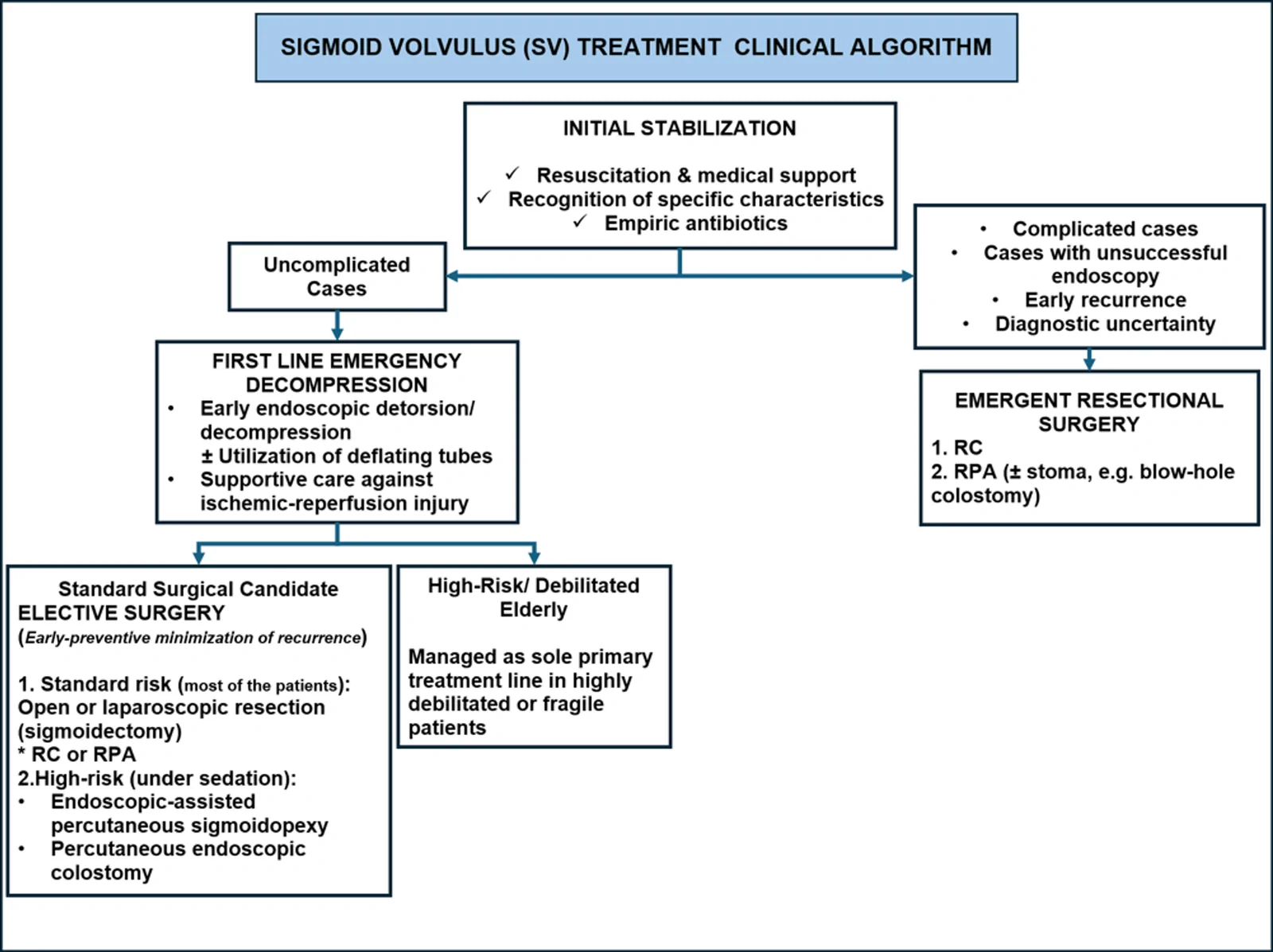
Management algorithm summarizing treatment options in relation to patient performance status and surgical risk. Treatment clinical algorithm for sigmoid volvulus. Sequential steps outline initial medical stabilization, emergency endoscopic decompression for uncomplicated cases, and subsequent elective or emergent surgical pathways based on patient risk profile and clinical presentation. The image was generated using Microsoft Excel. RC: resection with colostomy; RPA: resection with primary anastomosis

Uncomplicated Sigmoid Volvulus

In patients with uncomplicated SV, namely, who are not “in extremis,” flexible colonoscopy (rectosigmoidoscopy) under sedation is considered the diagnostic procedure of choice for assessing bowel viability. It also serves as the first-line, minimally invasive therapeutic intervention. The procedure involves advancing the endoscope to the distal point of the obstructed loop and gently attempting to pass it through the twisted segment [2,8,12]. Successful endoscopic detorsion was defined as passing through two points at the intestinal torsion site. Endoscopic decompression (deflating) is defined as the endoscope being able to pass only through the twist point on the anorectal side [1,2,5,7-10,12,27]. Reported success rates for endoscopic detorsion and decompression of the dilated sigmoid loop range from 55% to 92.5% [2,9,10,27,38-45]. Table 1 presents a summary of reported results after index endoscopic intervention or emergency surgery and the outcomes.

**Table 1:** Summary of clinical outcomes following index endoscopic intervention or emergency surgery. Comparative characteristics and therapeutic outcomes of patients with sigmoid volvulus across selected published studies. Clinical variables include patient sample sizes, mean age, endoscopic decompression success and recurrence rates, alongside surgical mortality outcomes. Yrs: years; n: number of patients; ND: not described; SV: sigmoid volvulus; TDT: transanal decompression tube; Pts: patients

Author, year (yrs)	Study design, period (yrs)	Institution, country	Patients (n)	Mean age, age range (yrs)	Endoscopic detorsion success rate (%)	Recurrence after endoscopic detorsion (%)	Mortality after surgery following endoscopy (%)	Mortality after emergent surgery (%)
Iida et al., 2017 [40]	Retrospective, 2008–2014	Muroran General Hospital, Japan	21	76 (54–90)	61.9% (13/21)	46.2% (6/13) ~11.8 months	-	12.5% (1/8)
Johansson et al., 2018 [38]	Retrospective, 2000–2016	Sahlgrenska University Hospital, Sweden	168	_	92% (403/438 admissions)	84% within ~58 days	3.3% (2/61)	13% (6/46)
da Rocha et al., 2019 [41]	Retrospective, 2009–2018	University de Lisboa Central, Lisbon, Portugal	52	83 (33–94)	86.4% (76/88 procedures)	81% (50% during 3 months after endoscopy)	0%	60% if necrosis, 14.3% if not necrosis (endoscopy)
Moro-Valdezate et al., 2022 [28]	Observational, 2000-2020	University Institute Valencia, Spain	92	81 (24–97)	87.8%	47.2%	9.1%	22.2%
Abdelrahim et al., 2022 [3]	Retrospective, 2016–2018	East Kent Hospital NHS, UK	40	82 (37–99)	92.5% (37/40)	80 % (within mean 86 days)	0%	33%, 30-day (2/6)
Nakamatsu et al., 2023 [42]	Retrospective, 2009–2022	Toyonaka Munic. Hospital, Osaka, Japan	70 uncomplicated	74.5 ± 11.6 and 75.8 ± 9.9	28.6% (20/70)	31 cases (7 detorsions +24 decompressions)	ND	ND
Satheakeerthy et al., 2024 [16]	Retrospective, 2000–2020	Waikato Hamilton Center, New Zealand	132	76 (18–97)	Rigid: 35%, flexible: 48% of 203 cases	48.8% (42/86) to 66.7% within 6 months	43 (32.6%) of all pts within 12 months, 8 deaths in 44 operated (18.1%)	
Hattori et al., 2024 [27]	Retrospective, 2008–2021	Sonoda Daiichi Hospital, Tokyo, Japan	69 uncomplicated	78 (37–101)	84% (58/69 : 11 needed surgery)	26.4% of TDT pts (14/53 )	2 deaths on septic pts at admission, 2 deaths among 3 with TDT who refused surgery	
Yamada et al., 2025 [44]	Retrospective, 2008–2022	Toyama Perfecture, Japan	52	40–90+	92.3% (48/52)	Of 45 pts with only endoscopic reduction: 35.6% within 2 yrs	ND	ND
Bock et al., 2025 [19]	Retrospective, 2019–2023	West General Hospital, Edinburg, UK	72	78 (43–95)	86.36% (57/66, discharged alive)	88% (50 of 57 readmitted with SV)	9 deaths: non-surgical candidates at admission	0% (0/6)
Watanabe et al., 2025 [43]	Retrospective, 2013–2024	Hoshigaoka Center, Hirakata, Japan	28	78.5 (47–95)	85.7% (24/28: 4 complicated)	62.5% (10/16, within 12 months)	8 deaths: 3 who did not undergo surgery, 5 who did (not disease-specific)	
Choi et al., 2026 [45]	Retrospective, 2005–2023	Yonsei University Seoul, South Korea	96	67.5 ± 17.9	80% (60/75)	36.66% (22/60 improved)	23.1% (3/13) among surgical pts, 22.2% (2/9) among non-surgical pts	

The success rate of endoscopic detorsion varies substantially, suggesting it may depend on factors such as the severity of the condition and the presence of complications [42]. Factors associated with a higher likelihood of successful endoscopic reduction include the absence of abdominal tenderness, a lack of previous open abdominal surgery, and the chronic use of laxatives [8]. Since the wider adoption of flexible endoscopy around 1998 and the exclusive utilization of flexible endoscopic decompression in subsequent years, procedure-related morbidity and mortality have remained low at 3% and 0.3%, respectively [12].

Following bowel detorsion, ischemia-reperfusion injury may aggravate intestinal dysfunction and potentially cause intestinal ulcers [5]. High intestinal fluid accumulation, electrolyte disturbances, and peritoneal exudates may also lead to serious adverse consequences. Therefore, effective supportive treatment, such as fluid resuscitation and antibiotic administration, is of paramount importance following successful colonoscopic derotation [5].

Although randomized controlled trials are lacking, current evidence supports the placement of one or more rectal decompression (deflating) tubes following successful endoscopic detorsion [27]. After confirming the viability of the colonic mucosa, identifying the twist site, and excluding other causes of intestinal obstruction such as rectosigmoid neoplasia, decompression tubes may be advanced proximal to the torsion point under colonoscopic guidance. These inserted tubes are typically 40-50 cm in length. They are left in situ for one to three days to maintain decompression and reduce the risk of immediate recurrence [2,10,12,14,27,33]. Concurrent bedside washing and irrigation of the colon using Ringer’s or normal saline solution may also be performed when indicated [10]. Additionally, endoscopic decompression serves as the initial treatment approach in pregnant women and pediatric patients [2,8].

Endoscopic detorsion or decompression may serve as the sole treatment for SV only during the first episode. However, it can be considered definitive for elderly, debilitated, or high-risk patients [10,38]. Nevertheless, recurrence remains a major limitation of conservative management. Although more than 20% of patients do not experience recurrence after an initial SV episode, reported mortality rates following non-operative treatment range from 9% to 36% [3,9,12]. For this reason, definitive surgical treatment is generally recommended after endoscopic decompression in medically fit patients.

Routine total colonoscopy is not required during the index SV episode unless there is clinical or radiological suspicion of underlying neoplasia [12]. However, in cases of diagnostic uncertainty, virtual colonography may provide additional diagnostic information and assist in excluding alternative pathologies [12].

Recurrence of sigmoid volvulus following endoscopic detorsion: As expected, successful endoscopic detorsion has a lower recurrence rate and prolongs the time to SV recurrence than endoscopic decompression alone [42]. However, successful endoscopic detorsion alone is associated with a substantial risk of recurrence over time. Reported recurrence rates range from 43% to 90% in adults and may approach 100% in pediatric patients. Conservative management is also associated with considerable mortality, with reported rates ranging from 15% to 42% [2,5,7,10,12,14,16,19,27,28,38]. In a recent study [9], the mortality rate was 62% among SV patients managed exclusively with endoscopic treatment, compared to 32% among electively operated patients. In approximately 13% of patients, recurrence occurs in the early period, within a few hours to days following successful detorsion or removal of the flatus tubes [7]. In a recent 20-year institutional series, nearly half of the patients treated non-operatively experienced recurrence. Two-thirds of these recurrences occurred within the first six months after the initial episode [16]. These findings highlight the limitations of endoscopic treatment as a definitive therapy and support the role of elective surgery in suitable candidates.

Surgical treatment following endoscopic detorsion of sigmoid volvulus: In the absence of randomized controlled trials, current consensus recommendations advocate elective colonic resection (sigmoidectomy) during the index hospitalization or within a few days (typically two to five days) following the first episode of SV and successful endoscopic detorsion [3,5,7,10,12,16,27]. Several studies suggest that early definitive surgery after initial endoscopic decompression is associated with improved outcomes and significantly lower mortality rates [3,38]. In a single-center cohort study comprising 168 SV patients, Johansson et al. [38] reported a mortality rate of 3.3% among patients undergoing planned surgery following successful decompression, compared with 13% among those requiring emergency surgery. Unfortunately, definitive surgery is frequently delayed in frail, elderly, high-risk surgical patients due to the increased risks associated with general anesthesia and major abdominal surgery [10,18].

In most series, resectional surgery has been the most frequently performed procedure in the acute setting [5,12,15,27]. The landmark study by Halabi et al. [15], which analyzed 63,749 colonic volvulus cases representing 1.9% of all bowel obstructions, illustrated the evolution of surgical practices in treatment over time. Accordingly, colonic resection was performed in 89% of cases managed surgically; mortality rates were reported as 9.44% for SV [15]. Technically, in elective surgery for SV, access is typically achieved through a low midline laparotomy or a left-sided abdominal incision. Non-oncological resection of the affected sigmoid loop is usually sufficient [12]. Resection may be completed either by primary colorectal anastomosis without mobilization of the splenic flexure, which is preferred, or by formation of a colostomy (Hartmann’s) when indicated [12]. More extensive resections, including subtotal or total colectomy, may be required, accounting for 16% of SV cases in the Halabi series. Indications include extensive colonic ischemia or unexpected necrosis, associated diffuse colonic atony, megacolon, or double volvulus [5,12,15]. Predictors of mortality after resection for SV include an age greater than 70 years and the presence of chronic cardiac, pulmonary, or renal disease, or a coagulation disorder. In advanced cases, the presence of peritonitis and bowel gangrene are the strongest predictors of adverse outcomes and mortality [15].

Minimally invasive techniques have increasingly been applied in the treatment of bowel obstruction, including selected cases of SV. However, the available experience remains limited and largely confined to specialized centers [6,12,46]. In the study by Halabi et al. [15], laparoscopic techniques were performed in only 3.7% of surgically treated cases of volvulus. With the laparoscopic approach, potential advantages include reduced postoperative pain, faster recovery of bowel function, shorter hospital stays, and possibly lower postoperative mortality in emergency surgical settings [6,12,15]. However, the principal challenge of laparoscopic operations in acute bowel obstruction, such as in SV, is the marked distension of intestinal loops. This distension substantially reduces the available working space and limits visualization and exposure of the operative field [6]. Consequently, laparoscopic sigmoidectomy is generally feasible only after successful decompression of the colon [13]. Furthermore, the friability of the distended intestinal wall increases the risk of severe iatrogenic injuries during trocar insertion and intraoperative manipulation [6]. Because of these significant technical limitations, careful patient selection is essential. Candidates should undergo a thorough preoperative assessment in specialized centers, including contrast-enhanced CT imaging of the abdomen and pelvis. Hemodynamically unstable patients and those with severe obesity are generally poor candidates for minimally invasive approaches. When laparoscopy is undertaken, open trocar insertion is recommended to minimize the risk of inadvertent bowel injury [6].

For patients considered high-risk surgical candidates, endoscopic-assisted percutaneous sigmoidopexy has emerged as a minimally invasive alternative [5,10,18]. By anchoring the sigmoid colon to the abdominal wall, sigmoidopexy restricts excessive colonic mobility and reduces the risk of recurrent torsion. Typically performed on the second or third day following successful decompression, the procedure can be performed under sedation without the need for general anesthesia [10,18]. Intentionally, patients are transferred to the operating theater, given prophylactic antibiotic coverage (e.g., ceftriaxone and metronidazole), and sedated in the lithotomy position [5,10]. The site of the sigmoid colon to be anchored to the peritoneum is located via colonoscopy and corresponds to the left lower abdominal quadrant [10]. Fixation is then achieved through the placement of suture anchors or endoscopic clips from the inside (transmural fixation) [10,18]. Although the method is generally well tolerated and effective in frail elderly patients, technical failure is common in patients with severe obesity, namely, a body mass index greater than 35 kg/m² [10]. Recent evidence suggests that despite clinical improvement and prevention of recurrent volvulus, some patients continue to experience transient colonic motility dysfunction and diffuse dilatation for several weeks following the procedure [18]. Recurrence rates after colopexy in various types of volvulus have been reported to range between 20% and 30% [12].

Percutaneous endoscopic colostomy has recently been proposed as another minimally invasive procedure for preventing recurrence after decompression, which does not require general anesthesia [12]. This approach is particularly attractive for debilitated patients, such as the elderly with severe comorbidities or institutionalized individuals who are poor candidates for major surgery and general anesthesia [12]. Although preliminary results are encouraging, the available evidence remains limited by small patient numbers and a lack of high-quality comparative studies.

Complicated Forms of Sigmoid Volvulus

Clinical findings, such as rebound tenderness, muscular rigidity, and the presence of gangrenous stool on digital rectal examination, strongly suggest bowel non-viability, perforation, or peritonitis, and constitute absolute indications for emergency surgery in acute SV [5,8]. Emergency intervention also becomes mandatory when bowel necrosis is identified during endoscopic decompression, when endoscopic detorsion fails, when detorsion is followed by recurrent SV, or when endoscopic treatment results in complications such as perforation [1,2,4,27]. Under these circumstances, urgent surgery is required both to eradicate intra-abdominal sepsis and to relieve the obstruction [8,12].

According to the clinical practice guidelines for colonic volvulus and ACPO from the American Society of Colon and Rectal Surgeons (ASCRS), summarized by Vogel et al. [14], urgent surgical intervention for SV was required in 5% to 22% of patients when endoscopic decompression was unsuccessful, while 5% to 25% of patients presented with gangrenous bowel at the time of diagnosis. Several studies have demonstrated a positive correlation between delayed presentation, prolonged preoperative intervals, which make endoscopic detorsion technically difficult or impossible, and the requirement for emergency surgery [8].

Colonic necrosis and generalized peritonitis remain the principal determinants of mortality in complicated SV [12,15]. At the same time, elderly patients and those with severe comorbidities require careful preoperative optimization. This process must balance the urgency of the intervention against the need for adequate resuscitation [7,12]. Crucially, surgery requires the correction of fluid and electrolyte abnormalities, the management of coagulation disturbances, and the restoration of hemodynamic stability.

Surgical options include resection of the affected bowel segment followed by colostomy, staged procedures involving colostomy and subsequent resection, or single-stage procedures involving resection and primary anastomosis [1,8,10,12]. The extent of the resection depends on intraoperative findings and the patient’s overall physiological status [12]. After a non-oncologic resection, bowel continuity may either be restored through a primary anastomosis (resection with primary anastomosis, RPA) or deferred by creating a Hartmann’s colostomy (resection with colostomy, RC) [1,4,12,32]. The optimal approach remains controversial owing to the lack of high-level evidence [4,32]. Therefore, treatment decisions should be individualized, taking into account both the patient’s overall status and the condition of the colon [4].

RC for SV, performed either as a Hartmann’s procedure or a double-barrel colostomy when technically feasible, remains the standard choice in most emergency cases [1,12,32]. Factors favoring stoma formation, which is the traditional approach for an unprepared, obstructed bowel, include advanced age, poor physiological status, and local risk factors, such as bowel perforation or peritonitis, questionable bowel viability, marked edema, and disproportionate diameters between the proximal and distal bowel segments [13,32]. The advantages of stoma creation include simplicity, shorter operative times, and the ability to monitor the downstream colonic segment in the acute postoperative period [12]. However, stoma-related morbidity in frail or elderly patients remains significant, and subsequent stoma reversal is often accompanied by substantial risks, or the procedure may even be abandoned altogether. Restoration of bowel continuity is generally performed electively after approximately 30 to 80 days, once the patient’s condition has improved and local inflammation has resolved.

Many researchers advocate RPA as the preferred surgical option for treating SV in stable patients without peritonitis, provided that a tension-free anastomosis can be achieved [12,13,30,32,47]. Potential benefits include definitive treatment in a single-stage procedure, the avoidance of stoma-related complications, potentially lower morbidity and mortality, and a shorter hospital stay. However, the presence of a markedly dilated and stool-laden proximal colon may increase the risk of postoperative anastomotic leakage. Consequently, restoring colonic continuity after resection remains controversial, even in the absence of gross contamination [12]. Factors associated with a higher incidence of major anastomotic failure in patients treated with RPA include acute anemia, perioperative whole-blood transfusion, and preoperative shock, particularly in those with gangrenous bowel [13]. The addition of a temporary proximal diverting stoma to an RPA case, such as a blow-hole colostomy in the transverse colon, has been proposed as a promising procedure to reduce the risk of anastomotic leaks and subsequent sepsis. This approach allows decompression while protecting the anastomosis, and it can usually be reversed after 15 to 30 days [4,13].

Several studies have evaluated single-stage resection combined with on-table colonic lavage. Although this technique facilitates anastomosis in an unprepared colon, it is associated with prolonged operative times and an increased risk of intraoperative contamination. These drawbacks make it less attractive for critically ill patients [12,47,48]. Furthermore, evidence from elective colorectal surgery suggests that mechanical bowel preparation is not essential for a safe anastomosis, and several studies have concluded that on-table lavage may not be necessary for emergency RPA in SV [13,47].

Comparative data regarding RPA and RC following emergency sigmoidectomy for SV remain limited [32]. A recent systematic review and meta-analysis, which included 10 cohort studies and one randomized controlled trial with 724 gangrenous SV cases, compared outcomes between RPA and RC [4]. Overall mortality was 15% in the RPA group and 19% in the Hartmann’s procedure group, while postoperative morbidity was slightly higher after RPA. However, none of these findings reached statistical significance. Coban et al. [13] reported an anastomotic leak rate of 6.3% among 25 patients undergoing RPA, whereas no leaks occurred among 22 patients treated with RPA combined with a protective blow-hole colostomy. Although these findings are promising, further prospective randomized clinical studies and well-designed, matched analyses are required to clarify the optimal operative strategy for complicated SV.

## Conclusions

SV is a surgical emergency associated with substantial morbidity and mortality. Accurate diagnosis and a comprehensive assessment of the patient’s physiological status can be challenging. This is particularly true in elderly patients and those presenting with advanced disease. Although several management aspects remain debatable, there is broad consensus that prompt, multidisciplinary evaluation and treatment are critical determinants of favorable outcomes. In uncomplicated SV, early endoscopic detorsion and decompression remain the standard initial treatment, followed by elective, definitive surgery, preferably during the same hospital admission. In contrast, emergency surgical treatment is required for complicated cases involving bowel ischemia, gangrene, and perforation-peritonitis. Emergency surgery is also indicated for failed endoscopic intervention, early recurrence, or diagnostic uncertainty. Current evidence supports that timely, definitive surgical treatment is associated with lower mortality and improved long-term outcomes. This benefit is especially pronounced for resectional procedures performed before the onset of severe complications. Nevertheless, further prospective studies are needed to clarify unresolved issues regarding the optimal timing and specific type of surgical intervention in both uncomplicated and complicated forms of SV.
